# Effect of 6-Min Mastication Test on Masticatory Function in Young Versus Older Adults: A Comparative SEMG Study

**DOI:** 10.1155/tswj/1506278

**Published:** 2025-09-15

**Authors:** Uzair Chilwan, Sahlah Shameer, Aysha Hanan, Radish Kumar Balasubramanium

**Affiliations:** Department of Audiology and Speech Language Pathology, Kasturba Medical College Mangalore, Manipal Academy of Higher Education, Manipal, India

**Keywords:** endurance, fatigue, mastication, older adults, surface electromyography

## Abstract

**Introduction:** Aging impacts masticatory and swallowing functions due to muscle atrophy, neural degeneration, and reduced endurance. This study evaluated age-related differences in masticatory muscle function using the 6-min mastication test (6MMT) with surface electromyography (SEMG).

**Methodology:** The study included two groups: healthy young adults (18–35 years) and healthy older adults (60 years and above). Muscle activity of the masseter and submental muscles was recorded using electrodes during mastication. Participants were instructed to chew bubble gum in synchronization with a metronome set at 80 bpm, with verbal reinforcement provided every minute.

**Results:** Results revealed significant age-related differences in the masseter muscle for all parameters and in the submental muscle for peak values. Older adults demonstrated reduced masticatory muscle performance, attributed to sarcopenia, neural degeneration, and increased noncontractile tissue. Within-group analysis showed progressive changes in submental muscle mean average and power values over the 6-min task, reflecting fatigue, while no significant differences were observed in masseter muscle parameters in healthy young adults, possibly due to a warm-up effect or compensatory motor unit recruitment.

**Conclusion:** The study demonstrated that masseter muscle activity significantly declines with age, with large effect sizes observed for all the parameters. Additionally, fatigue-related reductions in submental muscle parameters were observed across both age groups. These findings suggest that age-related and task-induced fatigue differentially affect masticatory muscles, highlighting the need for tailored interventions to support oral function in older adults.

## 1. Introduction

Mastication is the act of breaking down and grinding food which involves a coordinated effort among various oral structures including the lips, cheeks, tongue, palate, teeth, saliva, temporomandibular joint, and the masticatory muscles [1]. The primary masticatory muscles include the masseter, temporalis, medial pterygoid, and lateral pterygoid muscles. These muscles work in coordination to facilitate mandibular movements necessary for chewing and grinding food [2]. Continuous mastication and swallowing throughout a meal can be seen as an endurance activity as it not only demands sustained focus but also involves the continuous, repetitive use of various muscles at submaximal strength [3]. Alterations in the masticatory muscles with age lead to slower and less effective chewing [4], impacting their nutrient intake and overall health [5]. As endurance is essential for swallowing and mastication, easy fatigability can significantly impact this essential human activity [3]. Thus, preserving adequate masticatory function is essential for maintaining overall health [6].

Masticatory function over the years has been evaluated using surface electromyography (SEMG) (objective) and through their self-reported experience of chewing (subjective) [7]. SEMG is a noninvasive instrumental method used for analyzing bioelectrical activity generated from the muscles [8]. The application of electromyography to the study of masticatory muscles dates back to 1949, when Robert Moyers first used it to analyze muscle function in the jaw. Subsequently, in 1956, Greenfield and Wyke conducted foundational work on electrode placement for both surface and needle EMG in dental applications. These early investigations laid the groundwork for the development of SEMG as a valuable diagnostic and research tool in dentistry and oral rehabilitation [9]. It has been used to examine superficial muscles involved in chewing and swallowing and is often employed to assess the timing and intensity of muscle activation [10]. It involves placing electrodes on the skin over the target muscles to capture electrical signals, which are processed and displayed graphically on a computer as compound muscle action potentials [10]. The literature highlights the application of SEMG in monitoring the muscle activation patterns during swallowing tasks, assessing the maximum amplitude of muscle activity under swallowing conditions and furthermore examining the temporal relationships between swallowing kinematics and SEMG signals [11–13].

Multiple protocols are available in determining the endurance capacity of swallowing function. One such reliable test is the 6-min mastication test (6MMT), which assesses the endurance of continuous chewing. Studies have shown that factors such as pain and fatigue are better detected in isometric contractions [14]. This test has been found to have good reliability and reproducibility and is particularly useful in diagnosing masticatory issues in patients who report pain and fatigue during chewing [15]. The masseter, a key muscle in mastication, undergoes age-related atrophy, resulting in diminished occlusal force and compromised chewing efficiency. This progressive decline contributes to impaired eating function and elevates the risk of malnutrition [16]. Similarly, the geniohyoid muscle, which plays a vital role in hyoid bone elevation and laryngeal closure during swallowing, undergoes atrophy with age, contributing to an increased risk of aspiration in older adults [17]. As aging muscles are found to be more prone to fatigue due to an increased risk of sarcopenia, it will be interesting to assess endurance in the masseter and submental muscles during chewing across the age spectrum. Understanding these age-related changes has significant implications for dentists, speech–language pathologists, and geriatric medicine practitioners, who play a crucial role in managing oral and swallowing function. Identifying early neuromuscular decline can aid in developing targeted interventions to preserve mastication and swallowing efficiency, ultimately reducing the risk of malnutrition and aspiration-related complications in older adults. Therefore, this study is aimed at assessing and comparing the parameters of masticatory function in the masseter and submental muscles across young and older adults using SEMG. We hypothesized that older adults would exhibit significantly reduced masticatory muscle performance and endurance, as measured by SEMG parameters, compared to younger adults.

## 2. Material and Methods

### 2.1. Study Design

This study followed a cross-sectional design to complete the objectives of the study. The study was initiated after getting approval from the Institutional Scientific and Ethics Committee.

### 2.2. Participants

Fifty study participants were divided equally into two groups: healthy young adults (aged 18–35 years) and healthy older adults (aged 60 years and above). These age brackets were selected based on literature classifying 18–35 years as the period of peak muscle performance and 60 years and above as a phase where age-related muscle decline, including sarcopenia and reduced functional reserve, becomes more prominent [18–20]. Prior to commencement of the study, the pertinent medical histories and informed consents were obtained from the participants. Participants with any history of recent oral surgery, temporomandibular joint disorder, multiple missing teeth, and those who were using orthodontic appliances such as braces, retainers, or removable dentures were excluded from the study. Participants were verbally screened for symptoms of temporomandibular disorders using key questions adapted from the Axis 1 section of the Diagnostic Criteria for Temporomandibular Disorders (DC/TMD) Symptom Questionnaire [21].

### 2.3. Materials

Boomer chewing gum was used to assess the masticatory function and endurance over the course of 6 min. This chewing gum weighing 3.4 g per piece was chosen due to ease of availability and its feasibility of retaining the size and texture for a longer period of time due to continuous chewing.

### 2.4. Procedure

All the SEMG recordings were conducted during daytime hours, between 2:00 p.m. and 5:00 p.m., in a quiet environment at room temperature.

Skin preparation: The electrode sites were first cleansed using alcohol swabs (70% isopropyl alcohol) in a circular motion for 10 s to remove oil and debris and to reduce the skin impedance.

Electrode placement: Surface electrodes were placed bilaterally on the masseter and submental regions. For the masseter, electrodes were placed along the belly approximately at the midpoint between the zygomatic arch and the angle of the mandible. For the submental muscle, electrodes were placed centrally under the chin, targeting the suprahyoid muscle group. Although SNIAM guidelines do not provide direct recommendations for facial muscles, placement was done following anatomical landmarks and SEMG best practices described in previous literature.

Electrodes used: We used one-time disposable surface electrodes with a conductive surface area of approximately 1 cm^2^. The interelectrode distance was maintained at 2 cm. The maximum acceptable skin impedance was below 5 kΩ.

Participant posture: Participants were seated upright in a high Fowler's position, with their feet flat on the floor, knees at 90°, back supported, and head aligned with the Frankfort horizontal plane.

Task and repetitions: After a 40-s prechewing task (to form a single bolus), participants rested for 80 s. The main test, a 6-min mastication task, involved continuous bilateral chewing of two Boomer chewing gum pieces, in synchrony with a metronome set at 80 bpm. Verbal cues were provided every minute to maintain rhythm. Participants were allowed to swallow saliva as needed. Only one repetition of the 6-min task was conducted.

Equipment: The SEMG signal was recorded using the Chattanooga VitalStim Plus 4-channel unit (manufacturer: Chattanooga, United States), which includes a Bluetooth-enabled VitalStim Plus software interface (Version 1.0.12). Due to the therapeutic design of the device, the following data were not disclosed by the manufacturer: sampling rate, filter cut-off frequencies, input impedance, common mode rejection ratio, input range, and baseline noise.

Signal processing: Signal processing was done automatically using the VitalStim Plus software. The software output provided the following real-time parameters: mean average amplitude, peak amplitude, maximum voluntary contraction (MVC), and power values. The raw data were exported to Microsoft Excel for further statistical analysis.

### 2.5. Sample Size

Although an a priori sample size calculation was not performed, the target sample (*n* = 25 per group) was determined a priori by feasibility (time and resource constraints) and to align with typical sample sizes in SEMG studies of masticatory muscles [22–24]. Effect sizes with their 95% confidence intervals were calculated and reported alongside *p* values to provide an estimation of the magnitude and precision of observed effects. Raw data from both these muscles were obtained from each participant and were then transferred from the software to an Excel sheet, where the following parameters from both muscles were analyzed: mean average amplitude, peak amplitude, MVC, and power values.  Table 1 provides a summary of the definitions of various parameters obtained from the SEMG device.

### 2.6. Statistical Analysis

Jamovi Version 2.5.6 was used as statistical software to run descriptive and inferential tests. Descriptive statistics included the mean, median, standard deviation, and minimum and maximum values. A paired *t*-test was carried out to assess any differences in muscle function between 1^st^ and 6^th^ minutes in both groups. An independent sample *t*-test was used to check for age-related differences between healthy young adults and healthy older adults. For all statistical comparisons, effect sizes were computed alongside *p* values. Cohen's *d* was calculated for independent sample *t*-tests and Hedges' *g* for paired sample *t*-tests to account for small sample bias. Effect size interpretation followed the thresholds proposed by Zieliński and Gawda [9]: 0.1 = *small*, 0.3 = *medium*, and 0.7 = *large*. These values provide an estimate of the magnitude of differences observed between or within groups.

## 3. Results

The aim of the study was to check masticatory function and age-related changes in masseter and submental muscles using the 6MMT. Descriptive statistics were carried out for masseter and submental muscles, which have been summarized in Table 2.

From Table 2, it was observed that an evident difference was seen between healthy young adults and healthy older adults for all the parameters across both the muscles.

The Shapiro-Wilk test was carried out to check for normality (*p* < 0.005) where it indicated that data was normally distributed. Thus, paired sample *t*-test was carried out to assess the differences between 1^st^ and 6^th^ minutes among Group 1 and Group 2. The differences for the two groups along with effect sizes have been listed in Tables 3 and 4.

Table 3 highlights the results of the paired *t*-test in healthy young adults, where it was observed that no statistical difference was observed in all the parameters except the mean average and power value of the submental muscle. A medium effect was found for the mean average and power parameters of the submental muscle.

Results of the paired *t*-test (see Table 4) in healthy older adults revealed a statistical difference between 1^st^ and 6^th^ minutes for the mean average value in both the muscles and the power value in the submental muscle. A medium to large effect size was found for the parameters of mean average amplitude of both the muscles and the power value in the submental muscle.

Independent sample *t*-test was carried out to evaluate between-group differences.

Findings of the independent sample *t*-test from Table 5 revealed that a significant difference existed between healthy young adults and healthy older adults in all parameters of the masseter muscle. A large effect size (*d* > 0.7) was found for all parameters of the masseter muscle. However, no difference was found in the parameters of the submental muscles, except for the peak value. To further assess submental MVC, equivalence testing was conducted. The effect size was small (Hedges' *g* = 0.21, 90% CI [−0.26, 0.68]). Results of the equivalence test using bounds of ±0.30 failed to confirm equivalence (*p*_TOST_ = 0.716). Thus, while no difference was detected, equivalence between groups could not be statistically established.

## 4. Discussion

The present study is aimed at investigating the effect of aging on the masseter and submental muscles involved in masticatory function. The 6MMT using SEMG was used for assessing muscle activity and gaining insights into muscle physiology [25, 26]. Unlike recent literature focusing on crushing ability tests, the 6MMT using SEMG has been recommended for assessing masticatory endurance [25]. Hence, we utilized this tool for comparing and measuring sustained chewing performance among healthy young adults aged 18–35 years and healthy older adults aged 60 years and above.

Concordantly with the existing studies [18, 20] indicating that aging affects the masticatory muscle performance, our investigation hypothesized the negative impact of aging on mastication. The results of the between-group comparison indicated a significant difference in muscle activity performance between healthy young adults and healthy older adults, with a marked difference in the peak value of the submental muscle and notable variations in all the measured parameters of the masseter muscle. Alterations in masseter muscle functional parameters are evident in the context of sarcopenia, frailty, and aging [27].

With age, Type II fast-twitch muscle fibers, which are important for strength and quick movements, decrease in both size and number [19]. Consequently, when the masseter muscle was used for sustained mastication tasks, there was overall less muscle activation and efficiency in older adults, resulting in a lower mean average value of muscle activity, which was in line with a study conducted by Kim et al. [28]. A significant difference was found between both groups for MVC values in the masseter muscle, with a large effect size, thus indicating the clinical importance of age-related muscle decline in mastication. The MVC values in older adults have been reported to be 30% lower than in younger people, mainly because of age-related changes in muscle structure and nerve function [29]. Thus, the change in MVC value of the masseter muscle in older adults is likely caused by a combination of neural degeneration and muscle atrophy, which impairs their ability to generate and maintain high levels of contraction [30]. The difference in peak contraction values, along with a large effect size in our study, likely indicates an increase in noncontractile tissue in older adults, limiting their contracting efficiency for tasks that require sustained high force [31, 32].

In earlier studies, it was proved that there exists a decline in the peak value of submental EMG activity during both dry and liquid swallows in older adults [33, 34]. Our study findings build upon previous literature by demonstrating a significant difference in the peak values of the submental muscle between older and younger adults during sustained masticatory function over a predetermined period of 6 min. Therefore, submental muscle performance experiences age-related changes during swallowing function, which could potentially lead to decreased swallowing efficiency in older adults. Furthermore, these changes may be detected through SEMG before they manifest as clinical symptoms [35].

The current study exhibited differences between 1^st^ and 6^th^ minutes for parameters of mean average and power in the submental muscle within both the groups, which was in concordance with a similar study that evaluated SEMG activity in the Indian population [35]. However, all the parameters of the masseter muscle, along with peak and MVC values, did not show any difference between 1^st^ and 6^th^ minutes across both the groups, with the parameters exhibiting a low effect size. The possible reason could be the warm-up effect, where an increase in the temperature of the muscles would have resulted in an enhancement of maximal dynamic muscle performance [36]. Another reason could be the possibility of variation in muscle fiber recruitment. During localized muscle fatigue induced by repeated contractions, a reduction in electromyographic signal amplitude is typically observed due to diminished activity in fatigued muscle fibers. However, to sustain the required level of muscle tension, motor units compensate by increasing their firing frequency. This compensatory mechanism can lead to an overall increase in EMG signal amplitude as fatigue progresses, attributed to greater synchrony in motor unit activation [37].

These findings urge the dentists to implement strategies to maintain oral health and prosthetic support, whereas speech–language pathologists can design targeted exercises to enhance masticatory and swallowing efficiency. Geriatric specialists further can integrate nutritional and medical interventions to mitigate associated health risks associated with aging.

### 4.1. Limitations

This study has several limitations that warrant consideration. First, the sample size may have been insufficient to generalize the findings across broader populations, particularly when comparing masticatory function between younger and older adults. Additionally, the cross-sectional design limits the ability to assess changes in masticatory function over time, preventing insights into the progression of age-related muscle decline or potential improvements with intervention. The lack of longitudinal data further hinders the understanding of long-term changes in muscle function.

Another important limitation of this study is the use of the Chattanooga VitalStim Plus device, which is primarily a therapeutic SEMG device designed for biofeedback during neuromuscular stimulation rather than diagnostic-grade signal acquisition. While the device offers a user-friendly interface and real-time visual feedback, it does not disclose key technical specifications such as sampling rate, input impedance, common mode rejection ratio, filter settings, and baseline noise level. The absence of this information limits the precision and reproducibility of signal interpretation. Despite this, the device was selected for its accessibility and feasibility in clinical settings, and it provided reliable comparative data within the scope of this exploratory study.

Another limitation is the potential influence of subcutaneous tissue on SEMG readings, particularly in older adults, where higher fat percentages could have affected the accuracy of muscle activity measurements. The study also did not account for individual differences in lifestyle and nutritional health status, which could have impacted masticatory performance. Furthermore, only a few specific muscles, such as the masseter and submental, were assessed, potentially overlooking the roles of other masticatory muscles.

The lack of specificity in distinguishing which specific submental muscle is activated is a limitation while utilizing the SEMG data as there exist multiple activities of collective muscle groups [38]. Although submental MVC did not differ significantly between the groups, equivalence testing also failed to establish statistical equivalence. The wide confidence intervals highlight the need for larger samples in future work to clarify whether submental MVC is genuinely preserved with age or subject to subtle declines. Finally, extrinsic factors, such as variations in food consistency and bolus size during testing, were not standardized, which may have influenced muscle activation and impacted the results. Addressing these limitations in future studies will allow for a more comprehensive understanding of masticatory function and its age-related changes.

## 5. Conclusion

The present study demonstrates that age-related changes in masticatory muscle activity are more evident in the masseter muscle compared to the submental muscle group during sustained mastication. Significant between-group differences were observed in masseter mean average amplitude, peak amplitude, MVC, and power, all showing large effect sizes, indicating clinically meaningful declines in older adults. Within-group comparisons further revealed significant fatigue-related reductions in submental mean amplitude and power in both young and older adults, with medium to large effect sizes, suggesting these regions greater susceptibility to fatigue under prolonged chewing tasks. These findings highlight the importance of considering both age and muscle group when evaluating masticatory endurance and designing interventions. Future studies using diagnostic-grade EMG systems and larger sample sizes are recommended to validate and extend these findings.

## Figures and Tables

**Table 1:** Definitions of SEMG parameters.

**Parameters**	**Definition (unit)**
Average amplitude	Mean value of the signal's magnitude over a specified duration (*μ*V)
Peak amplitude	Highest magnitude reached by a signal during a measurement period (*μ*V)
Maximum voluntary contraction (MVC)	Greatest amount of force or torque that a muscle or group of muscles can voluntarily generate during a maximal contraction (%)
Power	Quantifies the cumulative electrical activity over a period of time (*μ*V × seconds)

**Table 2:** Descriptive statistics for masseter and submental muscles.

**Muscle**	**Parameter**	**Group**	**Mean**	**Median**	**SD**	**Min**	**Max**
Masseter	Mean average (*μ*V)	Group 1	41.1	38.4	14.5	21.2	79.6
		Group 2	31	28.4	14.9	10.3	57.1
	Peak (*μ*V)	Group 1	328	294	188	108	1013
		Group 2	183	193	97.4	45.2	314
	MVC (%)	Group 1	14	14	3.63	8	21
		Group 2	18.3	17	4.67	7	29
	Power (*μ*V × seconds)	Group 1	15,346	14,261	5373	7869	29,503
		Group 2	11,202	10,506	5859	1352	21,023

Submental	Mean average (*μ*V)	Group 1	24.6	22.9	8.87	5.6	45.3
		Group 2	21.4	23.4	9.7	7.9	49.3
	Peak (*μ*V)	Group 1	146	135	65.8	63.1	298
		Group 2	107	106	45	29	194
	MVC (%)	Group 1	19	18	6.46	9	34
		Group 2	20.2	22	5.71	10	28
	Power (*μ*V × seconds)	Group 1	9298	8703	3027	5127	16,574
		Group 2	7726	8645	3877	516	18,330

**Table 3:** Within group differences for healthy young adults.

**Muscle**	**Parameter**	**p** ** value**	**Effect size (Cohen's ** **d** ** )**
Masseter	Mean average	0.232	0.240
	Peak	0.937	−0.015
	MVC	0.146	0.294
	Power	0.436	0.155

Submental	Mean average	0.010	0.544
	Peak	0.375	0.177
	MVC	0.246	0.233
	Power	0.012	0.532

**Table 4:** Within group differences between healthy older adults.

**Muscle**	**Parameter**	**p** ** value**	**Effect size (Cohen's ** **d** ** )**
Masseter	Mean average	0.035	0.447
	Peak	0.210	0.257
	MVC	0.750	0.064
	Power	0.962	−0.009

Submental	Mean average	0.002	0.689
	Peak	0.053	0.407
	MVC	0.686	−0.081
	Power	0.002	0.696

**Table 5:** Between-group differences.

**Muscle**	**Parameter**	**p** ** value**	**Effect size (Cohen's ** **d** ** )**
Masseter	Mean average	0.014	0.710
	Peak	0.010	0.968
	MVC	< 0.001	−1.026
	Power	0.011	0.738

Submental	Mean average	0.224	0.345
	Peak	0.016	0.696
	MVC	0.458	−0.209
	Power	0.112	0.453

## Data Availability

The datasets generated during and/or analyzed during the current study are available from the corresponding author upon reasonable request.
